# Pathogenic Chytrid Fungus *Batrachochytrium dendrobatidis*, but Not *B. salamandrivorans*, Detected on Eastern Hellbenders

**DOI:** 10.1371/journal.pone.0116405

**Published:** 2015-02-19

**Authors:** Emma K. Bales, Oliver J. Hyman, Andrew H. Loudon, Reid N. Harris, Gregory Lipps, Eric Chapman, Kenneth Roblee, John D. Kleopfer, Kimberly A. Terrell

**Affiliations:** 1 Biology Department, James Madison University, Harrisonburg, Virginia, United States of America; 2 Gregory Lipps, LLC, Toledo, Ohio, United States of America; 3 Western Pennsylvania Conservancy, Pittsburgh, Pennsylvania, United States of America; 4 New York Department of Environmental Conservation, Albany, New York, United States of America; 5 Virginia Department of Game and Inland Fisheries, Richmond, Virginia, United States of America; 6 Smithsonian Conservation Biology Institute, Front Royal, Virginia, United States of America; University of Sao Paulo, BRAZIL

## Abstract

Recent worldwide declines and extinctions of amphibian populations have been attributed to chytridiomycosis, a disease caused by the pathogenic fungus *Batrachochytrium dendrobatidis* (*Bd*). Until recently, *Bd* was thought to be the only *Batrachochytrium* species that infects amphibians; however a newly described species, *Batrachochytrium salamandrivorans* (*Bs*), is linked to die-offs in European fire salamanders (*Salamandra salamandra*). Little is known about the distribution, host range, or origin of *Bs*. In this study, we surveyed populations of an aquatic salamander that is declining in the United States, the eastern hellbender (*Cryptobranchus alleganiensis alleganiensis*), for the presence of *Bs* and *Bd*. Skin swabs were collected from a total of 91 individuals in New York, Pennsylvania, Ohio, and Virginia, and tested for both pathogens using duplex qPCR. *Bs* was not detected in any samples, suggesting it was not present in these hellbender populations (0% prevalence, 95% confidence intervals of 0.0–0.04). *Bd* was found on 22 hellbenders (24% prevalence, 95% confidence intervals of 0.16 ≤ 0.24 ≤ 0.34), representing all four states. All positive samples had low loads of *Bd* zoospores (12.7 ± 4.9 S.E.M. genome equivalents) compared to other *Bd* susceptible species. More research is needed to determine the impact of *Batrachochytrium* infection on hellbender fitness and population viability. In particular, understanding how hellbenders limit *Bd* infection intensity in an aquatic environment may yield important insights for amphibian conservation. This study is among the first to evaluate the distribution of *Bs* in the United States, and is consistent with another, which failed to detect *Bs* in the U.S. Knowledge about the distribution, host-range, and origin of *Bs* may help control the spread of this pathogen, especially to regions of high salamander diversity, such as the eastern United States.

## Introduction

Chytridiomycosis, an infectious disease caused by the fungal pathogen *Batrachochytrium dendrobatidis* (*Bd*), has been implicated as a driving force of amphibian declines and extinctions worldwide [1]. Until recently, *Bd* was the only known *Batrachochytrium* species. However, a second species, *Batrachochytrium salamandrivorans* (*Bs*), was recently described in the European fire salamander (*Salamandra salamandra*) and was linked to declines in wild populations [2]. The newly described fungus is similar to *Bd* in that it can infect amphibian skin and cause chytridiomycosis [2]. However, *Bs* has a lower optimal growth temperature than *Bd* (10–15°C versus 17–25°C, respectively) [1,2], suggesting the newly described pathogen occupies a different niche. Interestingly, experimental infections reveal that the midwife toad (*Alytes obstetricans)* is resistant to *Bs*, despite the species’ known susceptibility to infection by *Bd* [2]. Despite the potential importance of *Bs* to global amphibian conservation, little is known about the distribution, host range, and origin of this fungus (but see [2–4]). In this study, we surveyed wild populations of the eastern hellbender (*Cryptobranchus alleganiensis alleganiensis*) for the presence of both *Batrachochytrium* species.

The hellbender is an aquatic salamander inhabiting cool (typically ≤20°C), highly-oxygenated rivers throughout the eastern U.S. and in parts of the Ozarks [5]. This species is the sole member of the family Cryptobranchidae in the western hemisphere, and its closest relatives, the Japanese and Chinese giant salamanders (*Andrias japonicus* and *Andrias davidensis*, respectively), are endangered in the wild. Although *Bd* has been detected on wild [6] and museum [7] specimens of *Andrias*, the susceptibility of these species to the resulting disease, chytridiomycosis, is unknown. Both the eastern and Ozark (*C*. *a*. *bishopi*) subspecies of hellbender have experienced widespread population declines in the last 30 years, likely due in part to habitat degradation and poaching [8–10].

Although *Bd* has been detected on hellbenders across a broad geographic area [11–16] and is believed to sometimes cause chytridiomycosis in captive individuals [17], the potential role of this pathogen in the species’ decline remains unclear. There is some evidence of seasonality in *Bd* infection of hellbenders, with prevalence being higher in cooler months of the year (March) compared to warmer months (May) [15]. Both captive and wild hellbenders are susceptible to skin disease that results in tissue necrosis, particularly on the ventral surface of the feet, but the cause of this condition is unknown [18]. Given that hellbenders are restricted to cool aquatic habitats (typically <20°C) [5], are commonly infected with *Bd* [11–16], and exhibit idiopathic skin disease *in situ* [5], it is a priority species for *Bs* testing. To our knowledge, *Bs* infection has not been reported for any North American amphibian or Cryptobranchid salamander [4].

We sampled populations of eastern hellbenders for the presence of *Bd* during routine population monitoring surveys from June 2012—July 2013. Because *Bs* was first reported before our samples were analyzed, we tested them for both pathogens simultaneously. Given the observation of presumed chytridiomycosis in captive hellbenders [17] and evidence that both eastern red-backed salamanders (*Plethodon cinereus*) [19] and southern mountain yellow-legged frogs (*Rana muscosa*) [20] lose weight when infected with *Bd*, we hypothesized that wild hellbenders infected by either *Batrachochytrium* species would exhibit poorer body conditions (as measured by a quantitative index calculated from weight and length) compared to uninfected counterparts. We further predicted that the prevalence of *Bd* infections in hellbenders would be lower during the warmest months of the year (i.e., July and August) when compared to animals sampled in early summer (i.e., June). We anticipated that knowledge about the prevalence and consequences of *Batrachochytrium* infection (particularly *Bs*) in eastern hellbenders could help inform the management of salamanders in this biologically-diverse region.

## Materials and Methods

### Hellbender sampling

Hellbenders (n = 88) were captured by dip net or net while snorkeling between June and August of 2012 and during July of 2013 from 17 sites in Ohio, Virginia, Pennsylvania and New York (n = 1–23 individuals per site). An additional three hellbenders (making n = 91 in total) were held in captivity at a regional fish hatchery, which has a flow-through system with water pumped directly from an adjacent stream that is known to be inhabited by hellbenders. Weight, length, life stage (adult vs. juvenile), and sex were recorded for each animal. Sampled individuals included 18 adult females, 34 adult males, 7 juveniles, and 32 individuals of unknown sex. The presence or absence of cloacal swelling (indicative of spermatogenesis) was used to determine the sex of adult hellbenders captured from mid July—early August. Individuals captured before that period were classified as unknown sex. Juvenile (total length ≤30 cm) [21] hellbenders cannot be sexed in the field. Hellbenders were rinsed with sterile Provasoli medium [22] in sterile plastic containers three times prior to sampling to remove dirt and other PCR inhibitors and to increase the likelihood that any detected *Batrachochytrium* was associated with the individual’s skin rather than the aquatic environment. New gloves were used between individuals. Each animal was swabbed with a sterile rayon swab (BBL CultureSwab, BD Diagnostics, Franklin Lakes, NJ, USA) ten times back and forth on their ventral side, and five times back and forth on each hand and foot. Swabs were stored on ice and frozen at -80°C within 24 hours of collection.

### Ethics Statement

All animal procedures were approved by the respective Animal Care and Use Committees at Smithsonian Institution (protocol #11–19) and at James Madison University (protocol #A13–12). Field research permits were obtained from the Virginia Department of Game and Inland Fisheries (permit #042343) and the Pennsylvania Fish and Boat Commission (permit #637). Sampling in New York (NY) was conducted directly by the NY Department of Environmental Conservation. Hellbender sampling in Ohio was conducted under letter permit from the Ohio Division of Wildlife issued to Gregory Lipps.

### Molecular Techniques

DNA was isolated from the swabs using the Mo Bio PowerSoil Kit (Mo Bio Laboratories, Inc. Carlsbad, CA) using the EMP protocol (http://www.earthmicrobiome.org/emp-standard-protocols/). Duplex real-time polymerase chain reaction (qPCR) was performed in triplicate using previously described methods [23] with a Bio-Rad CFX96 Touch Real-Time PCR Detection System (Bio-Rad, Hercules, CA). Negative and positive controls were included in each PCR run. In addition, the presence of PCR inhibitors was tested using internal controls [24] in one replicate well for each sample. *B*. *dendrobatidis* standard curves from 1 to 1000 zoospore genome equivalents (G.E.) were prepared from JEL 423 cultures maintained at R. Harris’ laboratory (James Madison University, Harrisonburg, VA, USA). *B*. *salamandrivorans* standard curves from 0.1 to 100 zoospore equivalents were prepared from cultures provided by An Martel (Ghent University, Merelbeke, Belgium.). A more sensitive standard curve was generated for *Bs* due to the lack of information about zoospore loads in wild amphibians. Samples were considered positive if all three wells contained more than one dilution-corrected zoospore equivalent of the pathogen. Samples that tested positive in only one or two wells were retested and considered positive if the pathogen was detected in at least one well in the subsequent run. Reported G.E. were corrected to account for the total amount of DNA isolated per swab.

### Statistical Analyses

Pathogen prevalence was calculated using the number of infected animals divided by the total sample size and included 95% Clopper-Pearson binomial confidence intervals. Chi-squared analyses were used to test the influence of sex and sampling month on the presence of infection. Body condition index (BCI) of each animal was calculated as the residuals from a regression of log-transformed body mass against log-transformed total length [25]. Six animals were dropped from this analysis due to improperly recorded data. The BCI of infected versus uninfected hellbenders was compared using a one-tailed Student’s t-test. Statistical tests were performed using SPSS V. 21(2012; Armonk, NY, USA). Results were considered significant at *P* < 0.05 and are reported as means ± standard error unless otherwise noted.

## Results

**Fig 1:**
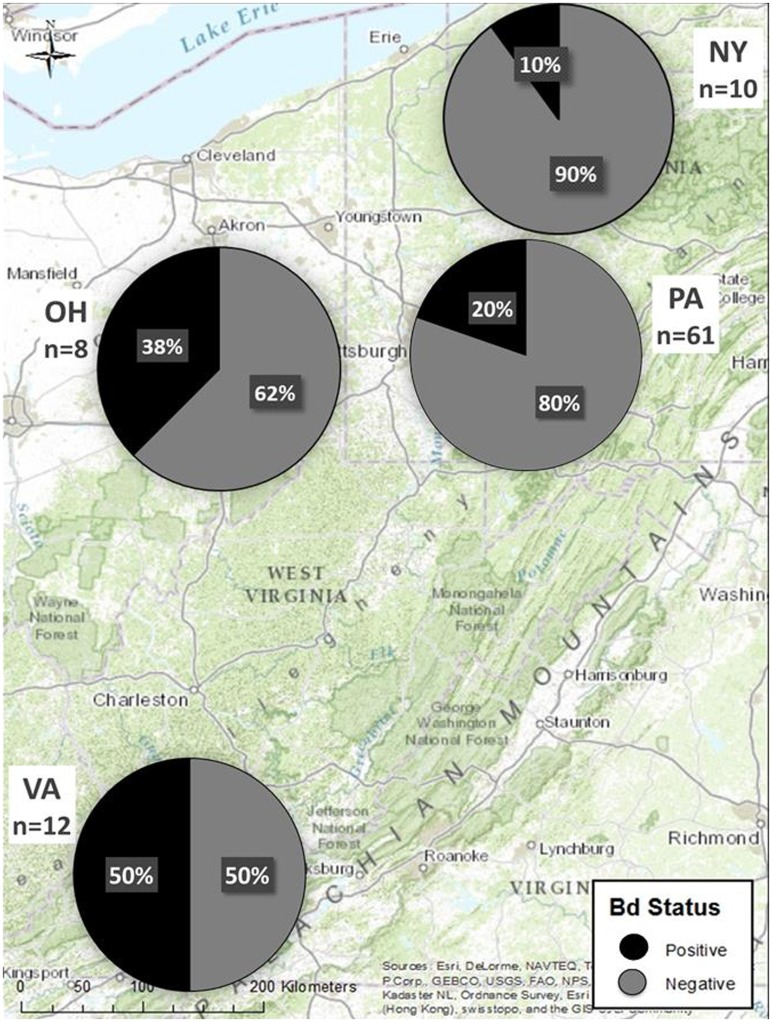
Map of *Bd* prevalence on eastern hellbenders. Hellbenders from all states sampled tested positive for *Bd*, but not *Bs*. Positive and negative proportions by state are indicated by pie charts.

**Fig 2:**
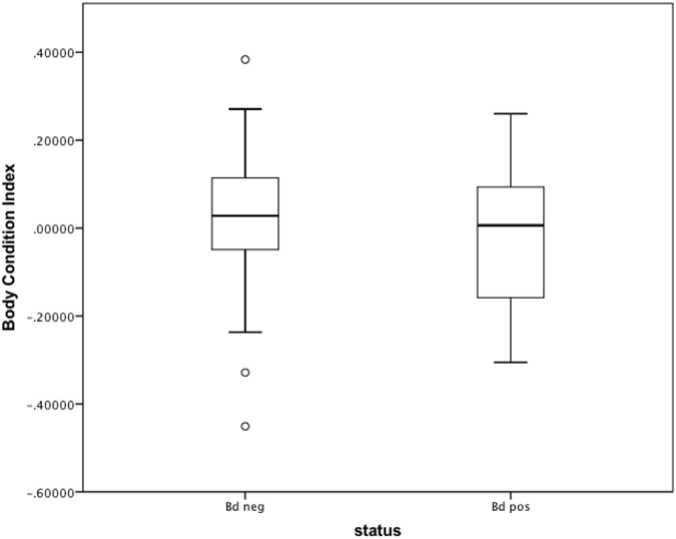
Body condition of *Bd*-positive versus *Bd*-negative eastern hellbenders. Body condition index of *Bd*-positive and *Bd*-negative animals did not differ (1-tailed t-test; t = -1.155, p = 0.176). The lower and upper edges of the boxes represent the first and third quartile, respectively. A bold horizontal line indicates the median value; vertical bars indicate the range of data, excluding outliers (indicated by circles).

Positive and negative qPCR controls indicated no evidence of inhibition or contamination, respectively. *B*. *salamandrivorans* was not detected in any sample (0% prevalence, 95% confidence intervals of 0.0–0.04). In contrast, *Bd* was detected in 13 (~72%) of the sites, representing all four states (S1 Table; Fig. 1). Overall *Bd* prevalence was 24%, with 22 samples testing positive (95% CI: 0.16≤0.24≤0.34). Of these samples, 17 tested positive initially, whereas five were negative in one well and subsequently rerun. In the second run, all five samples were positive in at least one well. All samples that tested positive contained *Bd* zoospore loads of ≤100, with a mean infection intensity of 12.7 ± 4.9 G.E. There was no influence of sex on infection probability (chi square _n = 52, df = 1_, p = 0.73), although 32 out of 84 adults could not be sexed because they were captured before the onset of seasonal spermatogenesis. No juveniles tested positive for *Bd* (0% prevalence, 95% CI: 0.0≤0.41), but life stage did not influence detection probability (chi square _n = 91, df = 1_, p = 0.12). Overall *Bd* prevalence in adults was 26% (95% CI: 0.17≤0.26≤0.37; S2 Table). The number of *Bd* positive animals differed by month (chi square _n = 91, df = 2_, p = 0.04), with higher prevalence in early summer (June) than mid-late summer (S1 Fig.). Body condition of *Bd*-positive animals was not significantly different from *Bd-*negative individuals (1-tailed t-test; t = -1.155, p = 0.176; N = 85; Fig. 2).

## Discussion

Despite the potential importance of *Bs* to global amphibian conservation, current understanding of this newly described pathogen is limited. To our knowledge, this study was the second to investigate the presence of *Bs* in North America [4] and the first to test for *Bs* in any Cryptobranchid salamander. Our findings yielded four important insights. First, our data suggest that *Bs* is not present in populations of hellbenders in the eastern United States. Second, our findings expand the known distribution of *Bd* in this host species, supporting the idea that the fungus is common (although not ubiquitous) throughout the hellbender’s range. Third, we detected low loads of *Bd* zoospores (≤100 G.E.) in all positive samples. In contrast, previous studies in hellbenders either did not quantify or did not report *Bd* zoospore load. Finally, we did not detect a relationship between *Bd* infection status and body condition, highlighting the need for empirical research to elucidate the consequences of *Bd* infection in hellbenders.

Although *Bs* does not appear to be present in eastern hellbender populations, more surveys will be needed to determine whether *Bs* is present within the U.S. These surveys should target aquatic or semi-aquatic salamander species that occupy thermal environments similar to that of the European fire salamander, such as mole salamanders (*Ambystoma* spp.), common mudpuppies (*Necturus maculosus maculosus*), red-spotted newts (*Notophthalmus viridescens viridescens*), or Pacific giant salamanders (*Dicamptodon spp*). Future surveys should also target potentially vulnerable geographic areas such as cities and regions with coastal ports, because they may be likely entry points for disease from pet trade [26]. Experimental infection trials will be necessary to determine which amphibian species outside of Belgium are susceptible to *Bs* infection and could serve as early indicators of the pathogen’s presence. This knowledge will also help identify species of concern for *Bs* related decline. It is also unknown whether *Bs* has alternative hosts. Future research should investigate potential non-amphibian hosts to *Bs*, such as crayfish [27] and waterfowl [28], which can carry *Bd* infections.

Although *Bd* has been detected on hellbenders from Arkansas [13], Georgia [15], Indiana [14], Missouri [11,13], Pennsylvania [29], and Tennessee [16], little is known about the individual and population-level impacts of this pathogen on hellbenders and other Cryptobranchids. Previous studies in hellbenders either have not measured or have not reported *Bd* zoospore loads. The infection intensities that we detected (12.7 ± 4.9 S.E.M. zoospore G.E.) were low compared to other amphibians [30] and far below the “~10,000 zoospore (per swab) threshold”, where declines in some amphibian populations become evident [31]. This suggests that hellbenders can limit colonization and reproduction of *Bd* on their skin. Determining how hellbenders limit *Bd* infection intensity in an aquatic environment may yield important insights for amphibian conservation against *Bd*.

We found no difference in body condition between *Bd*-positive and *Bd*-negative hellbenders. The low-level infections we found may not have been strong enough to cause negative growth effects. Alternatively, *Bd*-positive individuals with significantly deteriorating body condition could have died, either from infection or predation, and thus gone undetected. Importantly, hellbenders can consume large prey items, which could confound a weight-based body condition metric. Future hellbender studies should investigate alternative health metrics not based on mass, such as circulating leukocyte profiles [32] or fluctuating asymmetry [33]. Experimental infection of captive animals or mark-recapture studies of wild infected animals could also help elucidate how *Bd* affects hellbender fitness.

We detected a seasonal trend in *Bd* prevalence with a greater prevalence at the beginning of summer (S1 Fig.). This is consistent with past studies finding higher *Bd* prevalence and intensity during cooler temperatures [34]. However, because each site was visited opportunistically at only one time point, we cannot separate the effect of season versus site on *Bd* prevalence. Despite this caveat, these data suggest that seasonal variation in infection prevalence should be considered when monitoring hellbender populations for the presence of *Bd*. Hellbender population monitoring surveys are typically conducted during the summer months (primarily July-August) due to the difficulty of surveying in cold water conditions and high water levels in the spring. It will be important to monitor hellbenders during cooler months when they may be more susceptible to *Batrachochytrium* infection and chytridiomycosis or before and after cooler months by use of mark recapture.

Our results highlight the need for more research on the effects of *Bd* on eastern hellbenders. In addition, our limited knowledge of the distribution and host range of *Bs* emphasizes the need for caution against spreading this pathogen to potentially naïve regions. This caution is especially important for the eastern United States, a global center of salamander diversity [35]. Studies that examine the susceptibility of hellbenders and other U.S. salamanders to *Batrachochytrium* infection, coupled with regular monitoring of wild salamander populations, will facilitate the development of sound management strategies to protect these endemic amphibian species.

## Supporting Information

S1 Data Spreadsheet of all data used for statistical analyses. (XLSX)

S1 Fig Bd prevalence on eastern hellbenders by month (June-August). *Bd* prevalence was significantly higher for animals sampled in June (early summer) than for those sampled in July (mid-summer; chi square _n = 78, df = 1_, p = 0.02). Prevalence in August (late summer) was not significantly different from June (chi square _n = 36, df = 1_, p = 0.09) or July (chi square _n = 68, df = 1_, p = 0.81). Vertical error bars represent Clopper-Pearson 95% confidence intervals. Numbers below each month signify sample size for that month. Letters above error bars signify months with statistically significant differences in *Bd* prevalence.
(TIF)

S1 Table *Bd* prevalence for each of 18 total sampling sites across four states (N = 91). The location of each site is not presented in order to protect the hellbenders. VA Site 4 (n = 3) represents the captive animals sampled from the Buller fish hatchery.
(DOCX)

S2 Table Occurrences of *Bd* infection by sex and life stage of sampled hellbenders with Clopper-Pearson 95% confidence intervals. (DOCX)
