# Supplementary figures and images for: Pathogenic Chytrid Fungus Batrachochytrium dendrobatidis, but Not B. salamandrivorans, Detected on Eastern Hellbenders
Source: PLoS One. 2015 Feb 19;10(2):e0116405. doi: 10.1371/journal.pone.0116405 (PMC4335058; doi:10.1371/journal.pone.0116405)

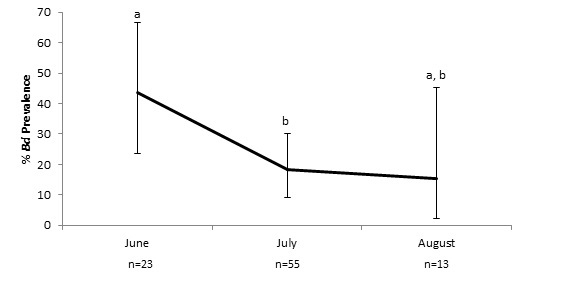

Supplement: S1 Fig — Bd prevalence was significantly higher for animals sampled in June (early summer) than for those sampled in July (mid-summer; chi square n = 78, df = 1, p = 0.02). Prevalence in August (late summer) was not significantly different from June (chi square n = 36, df = 1, p = 0.09) or July (chi square n = 68, df = 1, p = 0.81). Vertical error bars represent Clopper-Pearson 95% confidence intervals. Numbers below each month signify sample size for that month. Letters above error bars signify months with statistically significant differences in Bd prevalence. (TIF) [file pone.0116405.s002.tif]
