# Supplementary material for: Pathogenic Chytrid Fungus Batrachochytrium dendrobatidis, but Not B. salamandrivorans, Detected on Eastern Hellbenders
Source: PLoS One. 2015 Feb 19;10(2):e0116405. doi: 10.1371/journal.pone.0116405 (PMC4335058; doi:10.1371/journal.pone.0116405)
Supplement: S1 Table — The location of each site is not presented in order to protect the hellbenders. VA Site 4 (n = 3) represents the captive animals sampled from the Buller fish hatchery. (DOCX) [file pone.0116405.s003.docx]

**Table S1. *Bd* prevalence for each of 18 total sampling sites across four states (N=91).** The location of each site is not presented in order to protect the hellbenders. VA Site 4 (n=3) represents the captive animals sampled from the Buller fish hatchery.

| Site | N | % *Bd* prevalence | Clopper-Pearson 95% confidence interval |
| --- | --- | --- | --- |
| NY Site 1 | 10 | 10 | 0.3-44.5 |
| OH Site 1 | 5 | 40 | 5.3-85.3 |
| OH Site 2 | 3 | 33.3 | 0.8-90.6 |
| PA Site 1 | 1 | 100 | 0.025-1.0 |
| PA Site 2 | 3 | 33.3 | 0.8-90.6 |
| PA Site 3 | 4 | 50 | 6.8-93.2 |
| PA Site 4 | 3 | 33.3 | 0.8-90.6 |
| PA Site 5 | 4 | 0 | 0.0-60.2 |
| PA Site 6 | 23 | 13 | 2.8-33.6 |
| PA Site 7 | 3 | 33.3 | 0.8-90.6 |
| PA Site 8 | 1 | 0 | 0.0-97.5 |
| PA Site 9 | 13 | 0 | 0.0-24.7 |
| PA Site 10 | 5 | 60 | 14.7-94.7 |
| PA Site 11 | 1 | 0 | 0.0-97.5 |
| VA Site 1 | 2 | 0 | 0.0-84.2 |
| VA Site 2 | 2 | 50 | 1.3-98.7 |
| VA Site 3 | 5 | 80 | 28.4-99.5 |
| VA Site 4 | 3 | 33.3 | 0.8-90.6 |
| Mean |  | 31.65 |  |
