# Supplementary material for: Pathogenic Chytrid Fungus Batrachochytrium dendrobatidis, but Not B. salamandrivorans, Detected on Eastern Hellbenders
Source: PLoS One. 2015 Feb 19;10(2):e0116405. doi: 10.1371/journal.pone.0116405 (PMC4335058; doi:10.1371/journal.pone.0116405)
Supplement: S2 Table — (DOCX) [file pone.0116405.s004.docx]

**Table S2. Occurrences of *Bd* infection by sex and life stage of sampled hellbenders with Clopper-Pearson 95% confidence intervals.**

| Sex/Life Stage | *Bd*-positive  (n) | *Bd*-negative  (n) | % *Bd* Prevalence | Clopper-Pearson 95% CI |
| --- | --- | --- | --- | --- |
| Adult Males | 6 | 28 | 18% | 0.07≤0.18≤0.35 |
| Adult Females | 4 | 14 | 22% | 0.06≤0.22≤0.48 |
| Adults of Unknown Sex | 12 | 20 | 38% | 0.21≤0.38≤0.56 |
| Juvenile | 0 | 7 | 0% | 0.0≤0.0≤0.41 |
| All Adult  Samples | 22 | 62 | 26% | 0.17≤0.26≤0.37 |
| **Total** | 22 | 69 | 24% | 0.16≤0.24≤0.34 |
